# Agricultural wastes as a resource of raw materials for developing low-dielectric glass-ceramics

**DOI:** 10.1038/srep24617

**Published:** 2016-04-18

**Authors:** Satwinder Singh Danewalia, Gaurav Sharma, Samita Thakur, K. Singh

**Affiliations:** 1 School of Physics and Materials Science, Thapar University, Patiala-147004, India; 2 Department of Physics, School of Basic Sciences, Arni University, Kathgarh-176401, India

## Abstract

Agricultural waste ashes are used as resource materials to synthesize new glass and glass-ceramics. The as-prepared materials are characterized using various techniques for their structural and dielectric properties to check their suitability in microelectronic applications. Sugarcane leaves ash exhibits higher content of alkali metal oxides than rice husk ash, which reduces the melting point of the components due to eutectic reactions. The addition of sugarcane leaves ash in rice husk ash promotes the glass formation. Additionally, it prevents the cristobalite phase formation. These materials are inherently porous, which is responsible for low dielectric permittivity i.e. 9 to 40. The presence of less ordered augite phase enhances the dielectric permittivity as compared to cristobalite and tridymite phases. The present glass-ceramics exhibit lower losses than similar materials synthesized using conventional minerals. The dielectric permittivity is independent to a wide range of temperature and frequency. The glass-ceramics developed with adequately devitrified phases can be used in microelectronic devices and other dielectric applications.

Agricultural wastes could be used as resource materials in many engineering applications. In general, silica is a major constituent of the agricultural waste ash. It varies from 98–36 wt% in ash of rice husk, sugarcane leaves and corn husk etc^1^. In addition to silica, other metal oxides like CaO, MgO and K_2_O are also present along with some trace elements^2^. Rice husk after burning in air, produces highly reactive silica particles (usually nanosized)^3^. During this process, about 20% of biomass remains as ash, which contains different minerals^4^. These minerals can be amorphous or crystalline depending upon the conditions in which rice husk is burnt. On the other hand, sugarcane leaves ash (SCLA) have silica along with alkali and alkaline earth metal oxides as the major constituents^5^. Silica, derived from the waste, can be utilized as raw materials to form different high performance materials, such as glasses, refractories, capacitors, glass sealants, bioceramics, fibres and optical cavities, etc^6^,^7^,^8^. In addition to this, silica can also be converted to silicon after heating in the presence of activated charcoal. The formed silicon, after refinement, can be used in solar energy production and electronic chips^9^.

Formation of glasses and glass-ceramics from the agriculture waste have many benefits. It is environment friendly, economical and renewable source of high content silica. Moreover, it could be better and effective way to manage huge agricultural waste for producing different engineering materials. Additionally, during melt-quench process, the organic contents present in the waste materials convert in various gasses. These by-product gasses can be collected for further applications. Moreover, these gasses act as *in-situ* foaming agents and enhance the inherent porosity in the glasses and glass-ceramics. Inherent porosity in the glasses and glass-ceramics decreases the thermal conductivity, dielectric permittivity and density. On the other hand, it enhances the sensing and absorption of sound waves^10^. So, these glasses and glass-ceramics can readily find applications in the microelectronic devices, such as band-pass filters, dielectric resonant antennas and oscillators etc. For these applications, the material should have dielectric permittivity ~10 or above at room temperature with good thermal and mechanical stability. It also must have temperature and frequency independent behaviour in the microwave frequency region^11^. Silica and silicate based glasses could meet these requirements. High silica glasses are required for thermophotovoltaic system due to their good resistance to heat and thermal shock^12^. The disadvantage of the glasses and glass-ceramics derived from the agricultural wastes is the inability to vary the amount of different oxides, which prevents to tailor the properties according to the need and applications. This problem can be addressed by using different agricultural waste materials, which have different SiO_2_ content along with other elemental oxides as reported by Cornejo *et al*.^1^. So, the raw materials obtained from different agricultural waste ashes will provide necessary components for glass formation i.e. network former (SiO_2_) as well as network modifiers (K_2_O, CaO, MgO etc.) along with some trace elements. The trace elements may play important role to decide various properties of these glasses and glass-ceramics, since the role of trace elements is not known yet^1^.

The motivation of the present study is to develop low permittivity glasses and glass-ceramics directly from the ash of agricultural wastes instead of the conventional minerals. The structural and dielectric properties of formed glasses and glass-ceramics are investigated using different characterization techniques. The study is innovative and unique in the sense of glass and glass-ceramics derived from the agricultural waste, which leads to new opportunity to use the agricultural waste as resource for many engineering applications, such as microelectronic devices and other dielectric applications.

## Results and Discussion

Elemental analysis. The ICP analysis of the samples was done to know the amount of the elements present in the as-quenched samples. R-75 and R-100 contains mainly Si, while alkali and alkaline earth metals have negligible amount in these samples. Calculated composition of the samples from ICP analysis is given in Table 1. Besides alkali and alkaline earth content, the samples also contain very small amount of Cu, Zn and Fe. ICP results also revealed the presence of aluminium in substantial amount in these samples. Random variation of Al in the present samples comes from the crucible, since the samples are melted in Al_2_O_3_ crucible. Maximum Al_2_O_3_ content is found in R-50 sample. It contains maximum alkali and alkaline earth oxides, which decreases the overall melting point of this particular sample. So, the diffusion of Al^3+^ from the crucible is easier than other samples. The mol% of different minerals are different, as reported earlier, for the different food waste materials^1^. In the present case, the amount of different metals and metalloid oxides do not follow any trend as shown in Table 1. It is possible due to the local environmental effects on different agro-products.

The ICP results were further used to calculate the number of different structural units. Depending upon the number of different units, number of bridging and non-bridging oxygens was also calculated. In silicate glasses, the silicon-oxygen tetrahedron serves as the basic building block of the glass network^13^. These tetrahedra, linked at four corners, form a three dimensional network. Disorder in this structure is introduced by variable Si-O-Si angles, rotation of adjacent tetrahedra, oxygen vacancies (Si-Si) and peroxy defects (Si-O-O-Si)^14^. The modifier ions occupy the interstices in the network, which may reduce free volume of the glass structure. Sometimes, the occupancy of these modifiers is taken place in such a way, that free volume becomes zero or negative^15^. For every alkali (R^1+^) ion, there will be one non-bridging oxygen (NBO) and for alkaline (R^2+^) ion, there will be two NBOs. In the presence of the intermediate oxides like Al_2_O_3_, the estimation of NBOs and BOs becomes a little bit complicated due to its dual role as glass former and/or modifier. Alumina is a conditional glass former in the presence of silica^13^. It can form [AlO_4_] units, which incorporate alkali and alkaline earth metal ions to maintain the charge neutrality. R^1+^ and R^2+^ ions are consumed for the formation of AlO_4_ tetrahedra and decrease the NBOs. Al_2_O_3_ can act as a glass modifier also, depending on its local environment and amount within glass network^16^,^17^. By considering the contribution of all the components of the present samples, the NBOs and BOs are calculated. The calculated parameters are tabulated in Table 2. The number of NBOs and BOs can give fair idea about the transport properties of the material. More number of NBOs facilitates the easy motion of charged ions due to weak structure of such glasses. It may lead to enhance the conducting properties, which further influences the dielectric properties of the material.

**Figure 1:**
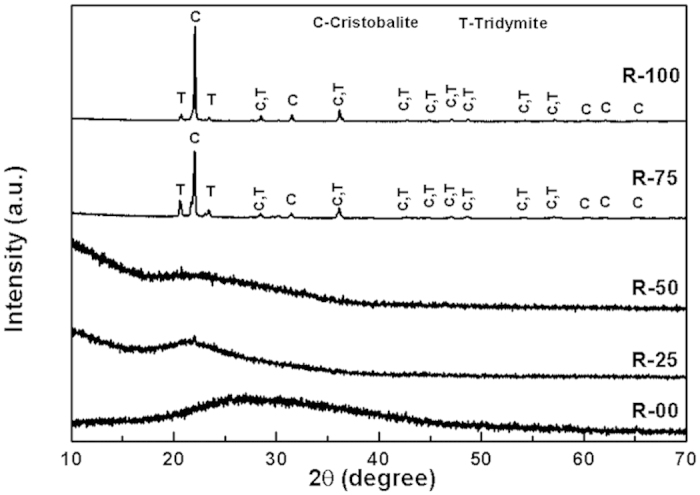
XRD patterns of the as-quenched samples.

X-ray diffraction. The XRD patterns of the as-quenched samples are shown in the Fig. 1. R-00, R-25 and R-50 exhibit amorphous nature as indicated by the broad XRD halos. A broad diffraction halo can clearly be seen around 17–35° in Fig. 1. It confirms the amorphous nature of the as-prepared samples. On the other hand, R-75 and R-100 are crystalline as evident from the sharp diffraction peaks. The XRD peaks of R-75 sample are indexed with cristobalite (ICDD no.-01-077-1317) and tridymite (ICDD no.-01-083-1339). On the other hand, R-100 sample is indexed with cristobalite (ICDD no.-01-075-0923) and tridymite (ICDD no.-01-076-0894). The volume fraction of these phases are calculated using direct comparison method^18^. The volume fraction of cristobalite and tridymite is 79 and 21%, respectively in R-75. The volume fraction of cristobalite increases in case of R-100 and becomes ~93%. The higher amount of SiO_2_ (as given in Table 1) may be responsible to increase the volume fraction of cristobalite phase as compared to tridymite phase in R-100 sample. The presence of alkali and alkaline earth metals may act as catalyst for the formation of tridymite phase^19^.

Silica, generally, melts above 1600 °C. So, R-75 and R-100, with high silica content, could not melt properly at 1550 °C and formed glass-ceramics. On the other hand, R-00, R-25 and R-50 glasses contain relatively higher amount of CaO, MgO, K_2_O and Al_2_O_3_. At certain concentrations, these oxides form eutectic mixtures and melt at substantially lower temperatures. For instance, in calcium aluminate glasses, simple oxides form eutectic mixture and melt with at lower temperature than melting point of individual elemental oxides. Similarly, sodium disilicate and silica eutectic mixture melts at ~800 °C^13^. Physical appearance of the R-75 and R-100 ceramics also supported the fact that with the increase in SCLA content, the melting of the composition became effective and easier. After visual analysis of the surfaces of R-75 and R-100, it was observed that R-75 pellet surface showed some liquid phase sintering. The decrease in amount of cristobalite at the cost of tridymite from R-100 to R-75 can also be explained on the basis of composition. R-75 melt has more modifying cation, hence, low viscosity as compared to R-100. Hence, the constituent ions could move easily and took their most likely positions to form thermally favourable tridymite phase. Based on the XRD results, it can be concluded that proper selection of the agricultural wastes could be used to tailor the properties of the glasses and glass-ceramics.

**Figure 2:**
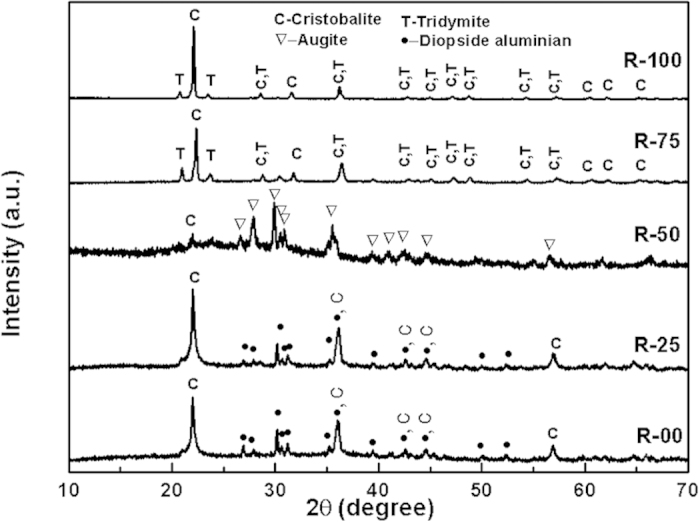
XRD patterns of the sample pelletsheat treated at 1000 °C for 10 h.

Figure 2 shows the x-ray diffraction patterns of the heat-treated pellets, which were used for dielectric measurements. After the heat treatment, R-00, R-25 and R-50, which were amorphous in as-quenched condition, transformed to glass-ceramics. On the other hand, R-75 and R-100, retained their glass-ceramic nature. However, full width at half maxima (FWHM) of the XRD peaks increases in the heat-treated samples as compared to the quenched samples (R-75 and R-100). Also, the diffraction peaks shift towards higher diffraction angle after the heat-treatment. The broadening and shift of peaks is more prominent for R-75 as compared to R-100. The broadening of the characteristic peaks indicates that during the heat-treatment, the as-quenched phases start dissolving and/or transforming to other stable crystalline phases. The volume fraction of the major phase i.e. cristobalite decreases to 76% for R-75 and 91% R-100, respectively. The decrease is accompanied by corresponding increase in the volume fraction of tridymite phase. Interestingly, R-00 and R-25 formed cristobalite, SiO_2_ (ICDD No.-00-039-1425) and diopside aluminian, Ca(Mg_0.5_Al_0.5_)(Al_0.5_Si_1.5_O_6_)(ICDD No.-01-080-0409) after heat treatment. However, cristobalite is a high temperature phase and forms at temperatures >1470 °C^20^,^21^. But, it has been reported that in the presence of alkali and alkaline earth content the cristobalite phase is formed even at low temperatures i.e. ~1000 °C^19^. It is well reported in the literature that the particle size of initial constituents can also play important role in the cristobalite phase formation^22^. R-50, with highest amount of modifying cations, shows distinguished XRD pattern than other glass-ceramics of the present series. Augite, Ca(Mg_0.70_Al_0.30_)((Si_1.70_Al_0.30_)O_6_) (ICDD No.-01-078-1392) is formed in this sample. Higher amount of modifying cations in R-50 hindered the formation of crystallised SiO_2_ phases. The nature and degree of crystallisation in these samples exhibits strong compositional dependence.

### FTIR and Raman spectroscopic analysis

**Figure 3:**
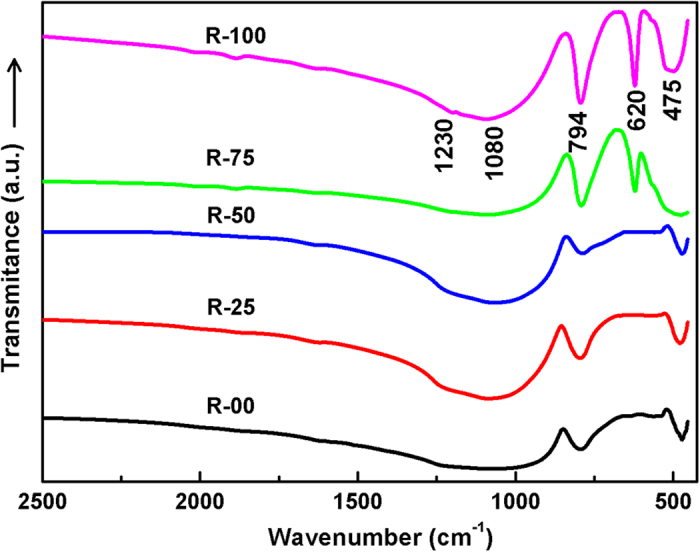
FTIR spectra of the quenched samples derived from agricultural waste ash.

Infrared transmittance spectra of all the as-quenched samples are presented in Fig. 3. The observed transmittance spectra of the present glasses and glass-ceramics can be explained as follows: (a) The broad band at 1080 cm^−1^ can be attributed to the Si-O-Si asymmetric stretching vibrations in the SiO_4_ units. Broadness of the band is due to the superposition of the IR-bands of the asymmetric vibrations of different type of Q-units of silica network. Q^4^ and Q^3^ units give rise to vibration bands at ~1100–1150 cm^−1^ and ~1050–1100 cm^−1^, respectively. Centre of the band at ~1080 cm^−1^ indicates abundance of SiO_4_ units with four and three bridging oxygen atoms^23^,^24^. The IR-band at ~794 cm^−1^ is due to the symmetric stretching of the Si-O-Si bonds of the SiO_4_ tetrahedra. (b) The bands around 475 cm^−1^ are mainly due to the Si-O bending vibrations^25^,^26^. The bending vibrations of Al-O bonds also occur within this wavenumber range^27^. (c) The band around 620 cm^−1^, which is present only in R-75 and R-100 glass-ceramics, is the characteristic band of cristobalite^26^. The presence of this band, only in these samples, is also well supported by XRD results, which clearly indicates the presence of cristobalite phase in both the glass-ceramics. This band becomes sharp and more intense in R-100 as compared to R-75, which indicates the higher volume fraction of cristobalite phase. (d) IR band at ~565 cm^−1^ corresponds to stretching vibrations of Si-O-Al molecules^28^. The observed bands exhibit a red shift for all the samples. It can be attributed to the weakening of the structural units due to increase in the NBOs^29^,^30^.

**Figure 4:**
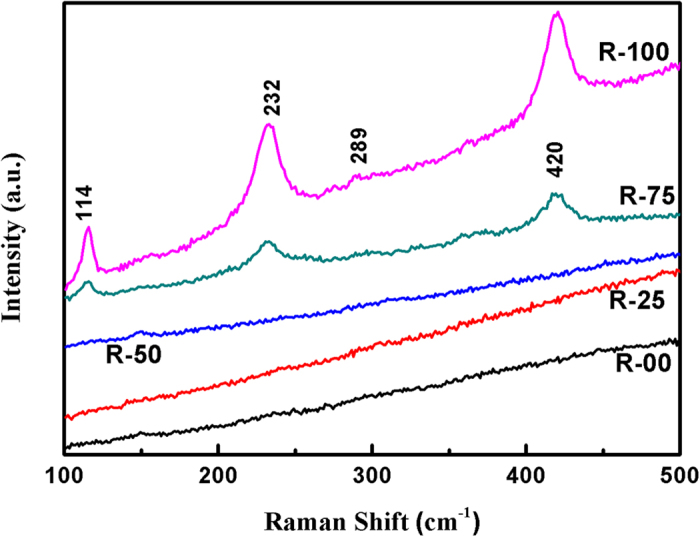
Raman spectra of the quenched samples derived from agricultural waste ash.

Figure 4 shows the Raman spectra for present glasses and glass-ceramics. Raman spectra mainly show three bands for R-100 and R-75 glass-ceramics i.e. ~114, 232 and 420 cm^−1^, respectively. On the other hand, R-50 glass shows a very feeble band ~148 cm^−1^. In addition to this, it is observed that the spectra of R-100 and R-75 exhibit higher band intensity at higher wavenumbers. It can be related to either thermal background or to the possible luminescence from the material^31^. The bands exhibit slight variation due to formation of NBOs and change in structural units^32^. In general, Raman bands in silica and silicate glasses fall in three regions namely, near 1060 and 1200 cm^−1^ with weak intensity, ~800 cm^−1^ with medium intensity and ~430 cm^−1^ with maximum intensity^33^. Disturbed short range order due to glass modifier ions may suppress the intensity of Raman bands. Hence, the low intensity bands disappeared and only high intensity bands could be visible in the present samples. The bands at ~114, 232 and 420 cm^−1^ are attributed to symmetric stretching of Si-O-Si bond in cristobalite (SiO_2_) phase^34^,^35^. These Raman bands are asymmetric as indicated by the deviation from pure Gaussian curve. It can be attributed to the existence of more than one type of the [SiO_4_] structural units^36^. Also, it can be clearly observed that the intensity of Raman peaks decrease sharply with SCLA addition. Raman bands in R-75 spectrum have low intensity than bands in R-100 spectrum. While, further addition of SCLA results into complete diminish of the Raman bands. It may be possible that R-00, R-25 and R-50 are also Raman active but the peaks are too weak to be measured. When cristobalite phae transforms to another phases, then low wavenumber raman bands at 114, 232 and 420 cm^−1^ diasappear^37^. Hence, Raman spectra R-00, R-25 and R-50 glasses could not show any observable Raman bands due to absence of cristobalite phase. Generally, NBO-Si bond should exhibit the Raman band near 900 cm^−1^ 38. However, in the present case, amount of alkali content is low, that’s why band at this position could not be observed (as shown in Fig. 4).

### Dielectric permittivity

At a constant temperature, the dielectric response of a material changes with the applied filed in accordance with the following equation:


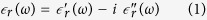


**Figure 5:**
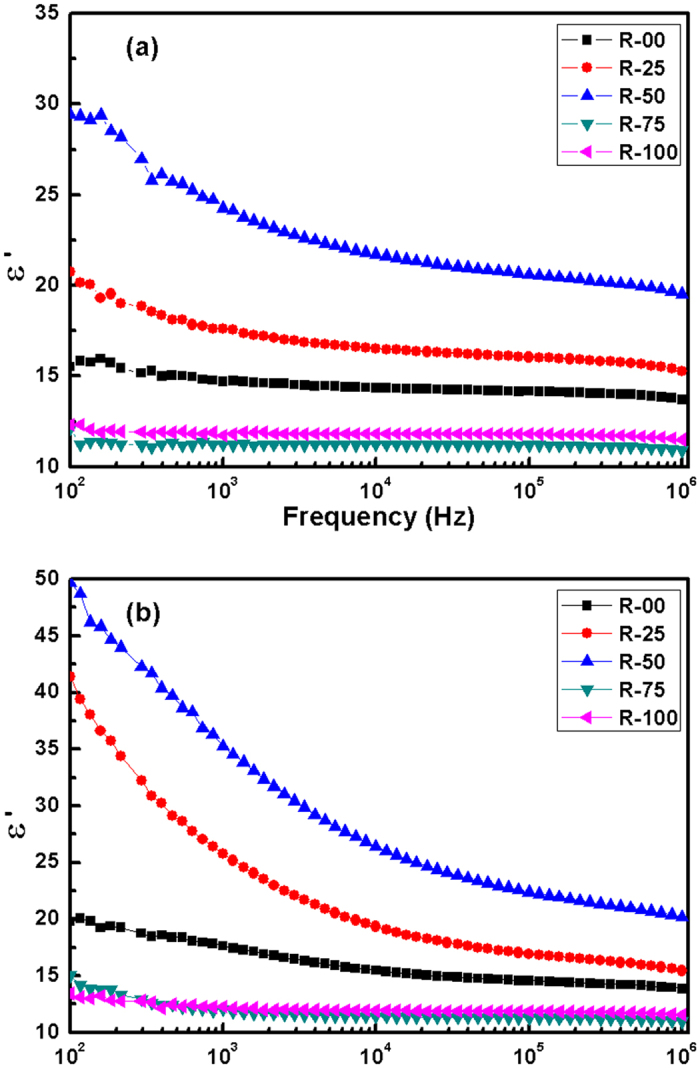
Variation in dielectric permitivity with frequency for the glass-ceramics at (**a**) 250 °C (**b**) 350 °C.

Here, the symbols have their usual meanings. The variation of dielectric permittivity (ε′) versus frequency for present glass-ceramics is presented in Fig. 5. The dielectric data clearly shows the low tanδ (losses) in the glass-ceramics. Secondly, the dielectric permittivity of the samples is nearly independent of frequency from room temperature to 250 °C. At higher temperatures (>350 °C), dielectric permittivity increases sharply, particularly for lower frequency region. R-50 exhibits maximum ϵ′ followed by R-25, R-00, R-100 and R-75. Dielectric permittivity of present samples is nearly proportional to the modifier content. It can be co-related with the highly mobile nature of the alkali and alkaline earth ions. Conduction in the glass-ceramics is dominated by the hopping of the alkali ions. Hopping of ions becomes easier with higher defects, which act as hopping sites. This may be the reason for highest dielectric permittivity of R-50 (higher NBOs) as compared to other samples. R-100 and R-75 contain very low modifier cations, which leads to lower dielectric permittivity. Similar high silica compositions, when synthesized by conventional minerals, show low dielectric permittivity, but higher losses than present samples^39^. Additionally, the higher volume fraction of crystalline phases may increase the electronic conduction as compared to ionic conduction, which could be responsible to decrease the dielectric permittivity. Basically, the decreased ionic motion reduces the dielectric permittivity^40^. As mentioned in XRD section, R-50 formed augite phase, which has monoclinic prismatic structure. It is disordered structure as compared to tetragonal cristobalite and hexagonal tridymite phases. More disordered structure makes the system relatively more polar in the presence of the applied field. R-00 and R-25 formed diopside, which is monoclinic and closely related to augite. It makes these samples to have higher dielectric permittivity as compared to ordered R-75 and R-100 samples. Increase in dielectric permittivity of R-00, R-25 and R-50 at lower frequencies is usual behaviour and can be explained on the basis of Maxwell-Wagner dielectric theory^41^. At lower frequencies, the space charge polarisation plays a dominant role and increases the dielectric permittivity. This effect predominates at high temperatures, where the thermal vibrations facilitate the ionic motion due to increased mobility. These mobile charge are accumulated at the surface of the material and enhances the dielectric permittivity^42^. Therefore, at low frequencies and higher temperatures, the dielectric permittivity of the glass-ceramics increases.

**Figure 6:**
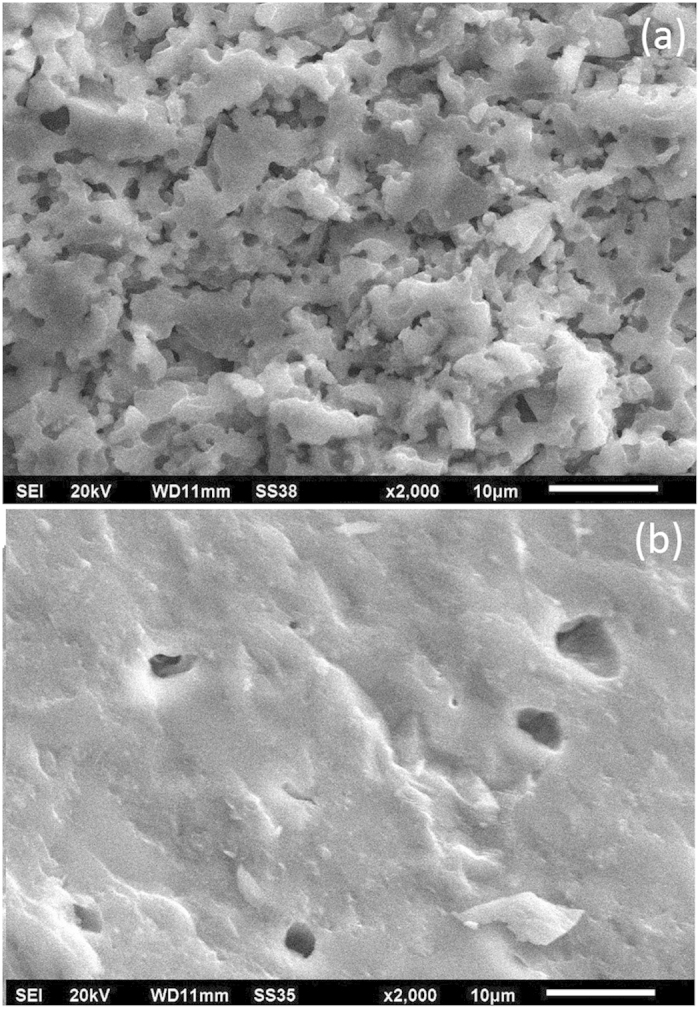
SEM images of the fractured surfaces of the pellet of (**a**) R-75 glass-ceramic (**b**) R-100 glass-ceramic.

**Figure 7:**
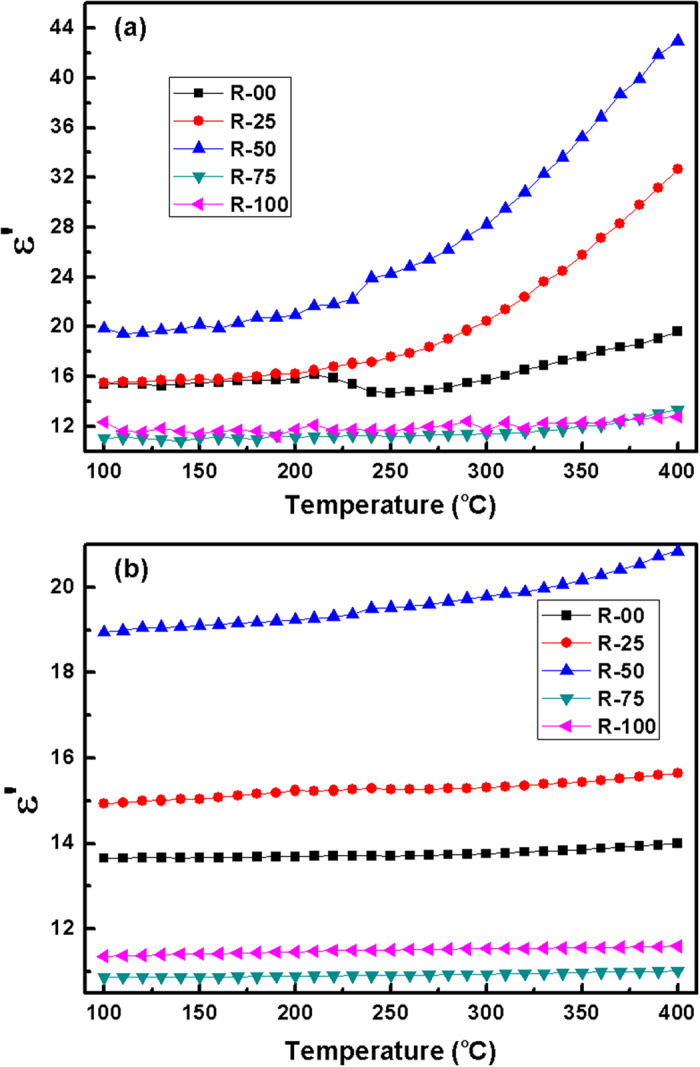
Variation in dielectric permittivity with temperature for the glass-ceramics at (**a**) 1 kHz (**b**) 1 MHz.

Interestingly, ϵ′ of R-75 is less than that of R-100, despite of its higher alkali content. It can be explained on the basis of the porosity of these glass-ceramics. SEM images of the fractured surfaces of the pellets of these glass-ceramics indicate more porosity in R-75 than R-100 sample (Fig. 6). Air is trapped in these pores and leads to decrease the dielectric permittivity^43^. The variation in dielectric permittivity with temperature at selected frequencies is shown in Fig. 7. The temperature independent behaviour of dielectric permittivity of these glass-ceramics at high frequencies (1 MHz) is clearly observed as shown in Fig. 7(b).

**Figure 8:**
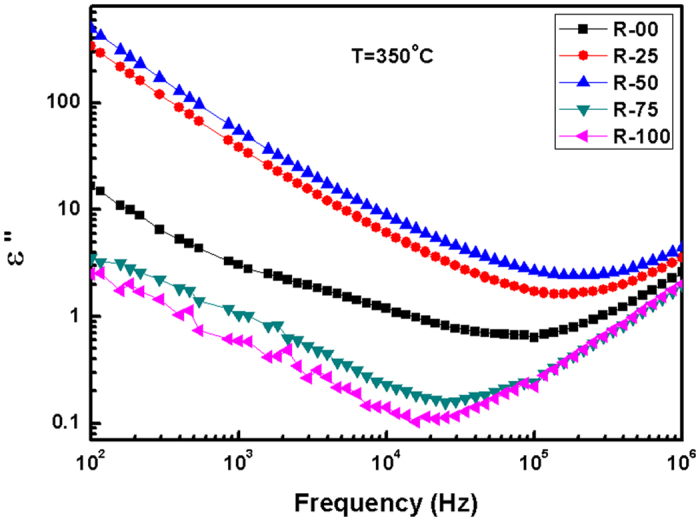
Dielectric loss of the R-50 glass-ceramic with frequency at 350 °C.

**Figure 9:**
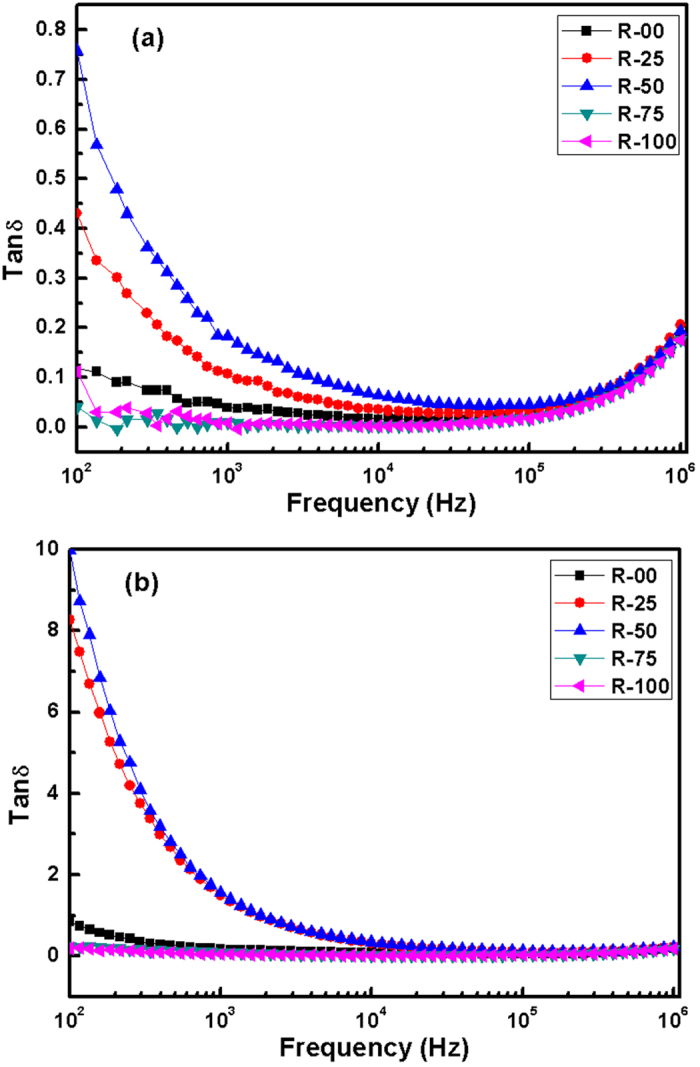
Tanδ vs frequency plots for the glass-ceramics at (**a**) 250 °C (**b**) 350 °C.

Figure 8 represents the variation in dielectric loss (ϵ′′) versus frequency at 350 °C. Absence of any peak confirms that the dielectric permittivity of these glass-ceramics is mainly due to conduction processes. Loss tangent (tan δ) versus frequency at various temperatures is shown in the Fig. 9. tanδ represents the dissipation of energy due to various physical processes (relaxation). These losses are associated with the crystal imperfections, porosity, defects and electrical conduction etc. Glass-ceramics always have higher losses than crystalline counterparts of similar materials due to higher defects^44^. In the present samples, losses are less than earlier reported glasses formed by conventional minerals^45^. tanδ increases at lower frequencies and also with temperature. It can be seen that increase in the tanδ at lower frequencies is prominent in R-00, R-25 and R-50. However, it remains almost constant for R-75 and R-100. It again reflects the dominance of modifier concentrations on dielectric properties as well as basic structure. The low dielectric permittivity of the present samples, along with temperature and frequency independence in wide range, make them good candidate for microelectronic applications.

### Electric modulus

The electric modulus (M*) can be expressed in terms of the complex permittivity (ϵ*) of the material as follows:


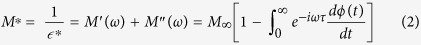


where, the function 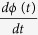 presents the time evolution of the electric field inside the dielectric material. The real and imaginary parts of the electric modulus can be given as:


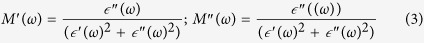


**Figure 10:**
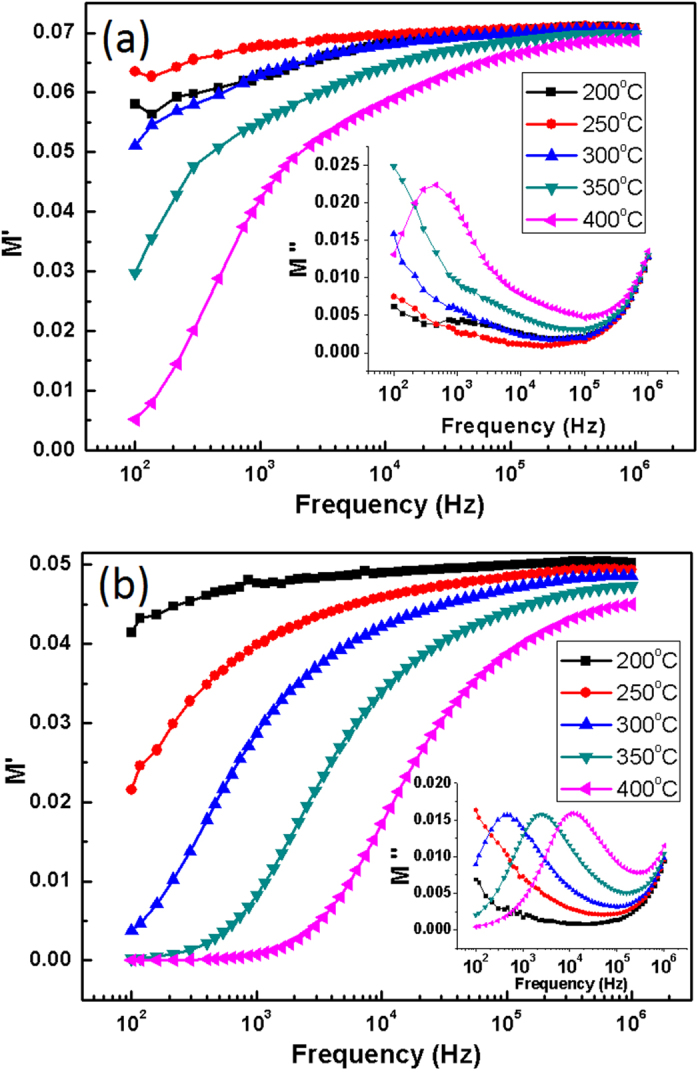
Real part of the relative modulus variation with frequency at selected temperatures for (**a**) R-00 (**b**) R-50. Inset of the figures show the imaginary part of relative modulus of repective samples.

Modulus formulation is employed to nullify the electrode effects. Representation of dielectric data in this form provides additional insight into the relaxation behaviour of the glasses^46^. The variation in *M*′(*ω*) and *M*″(*ω*) with frequency at selected temperatures is given in representative Fig. 10(a,b), respectively. For all the samples, the *M*′(*ω*) curves show the declination towards zero at low frequencies, indicating the suppression of electrode effects^47^. It confirms that the dielectric permittivity (previous section) at low frequencies is also contributed by the electrode polarisation. As for as *M*″(*ω*) is concerned, a universal feature of increasing *M*″(*ω*) at very high frequencies (>10^5^ Hz) and very low frequencies (<10^3^ Hz) is observed for all the samples. These features might be the residual parts of the relaxation peaks and might appear as complete peaks, if the measurements would have been taken above 10^6^ Hz and below 10^3^ Hz also. This hypothesis can be supported by the general fact that in glasses, M″ curve shifts to higher frequencies with increase in the temperature. A close observation of M″ curve for R-00 exhibits a relaxation peak at 400 °C, whose appearance might be a consequence of the peak shifting towards higher frequencies. Besides these observations, clear relaxation peaks can be seen for R-25 and R-50 glasses at selected temperatures. These peaks shift to the higher frequencies with increase in temperature. The position of these peaks separates the long range mobility region of ions (low frequencies) and the spatially confinement of the ions (high frequencies).

### AC conductivity

From the dielectric loss and the real part of the dielectric permittivity, the value of ac conductivity is obtained using the following relation:


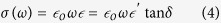


**Figure 11:**
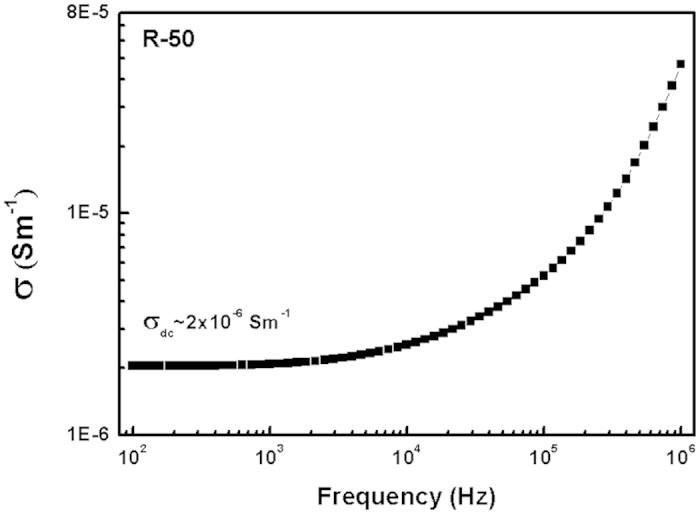
Conductivity vs frequency curve for R-50 glass-ceramic at 400 °C.

At lower frequencies, the conductivity is nearly constant for all the samples. At higher frequencies, the conductivity increases sharply and follows the universal power law. The conductivity curves were fitted using power law as follows:


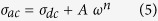


where, A is a constant, σ_dc_ is the dc conductivity and *n* represents the degree of correlation between mobile ions. For present samples, the value of *n* remains less than 1 at 400 °C, which indicates dominance of the ionic conduction in the samples. Figure 11 shows the representative conductivity curve of R-50 at 400 °C. Oxide glasses are generally ionic conductors^48^. Hence, in the present glass-ceramics, the conductivity was expected to increase with alkali and alkaline earth metals content. Similar trend are observed in the present glass-ceramics. R-50 exhibits maximum conductivity followed by R-25, R-00, R-75 and R-100. Generally, ionic conductivity increases with disordering, while electronic conductivity decreases. In present samples, even after heat treatment, R-50 sample is more disordered than other samples. The higher conductivity is obtained for R-50 sample i.e. 2 × 10^−6^ Sm^−1^. The value of conductivity is higher as compared to the similar system synthesized by the conventional materials^49^.

Moreover, the conductivity shows both temperature and frequency dependent behaviour. With the increase in the temperature, the conductivity of the samples increases. This indicates that the conductivity in the present samples is a thermally activated process^50^.

The agricultural wastes can be used as a resource to for, the different glasses and glass-ceramics. The addition of sugarcane leave ash in rice husk ash promotes the formation of glasses due to effective eutectic reactions among different components of agricultural waste ashes. The dielectric losses (tanδ) are observed less than 0.1 in R-75 and R-100 glass-ceramic samples. The dielectric permittivity of the glass-ceramics R-75 and R-100 samples is independent to wide range of temperature and frequency. The glass-ceramics developed with controlled heat-treatment can be used in microelectronic devices and other dielectric applications.

## Methods

Rice husk was obtained from a rice mill and sugar cane leaves were taken from an agricultural farm. Both were washed with water to remove adhering soil and dust and then burnt in closed vessel in air to convert into ash. Five samples with different weight percentage of rice husk ash (RHA) and sugarcane leaves ash (SCLA) were taken.

### Sample preparation

Compositions (*x*) *RHA*-(*100-x*) *SCLA*, where *x* = *0, 25, 50, 75* and *100* by *wt%* (accordingly they are labelled as R-00, R-25, R-50, R-75 and R-100) were ground and mixed in agate mortar-pestle for homogenization. These homogeneous mixtures were calcined at 1000 °C. The calcined powder was again ground in agate mortar pestle to get very fine powder. Then, pellets of this powder, with small amount of binder (PVA), were prepared using hydraulic press. The applied pressure was 10 kNcm^−1^. Afterwards, these pallets were kept in programmable furnace for melting 1550 °C. The melts were tried to quench on the thick copper plates, but due to very high viscosity it could not be poured on the copper plates. For R-00, R-25 and R-50 compositions, the melt was in the form of a rigid and foamy semi-solid material. Besides this, R-75 and R-100 compositions could not melt completely and retained their pellet form.

### Characterisations

After melting, the composition of the samples were determined by Inductively Coupled Plasma Mass Spectrometer (Thermo Fisher Scientific, Element XR) with quantification limit better than 1 ppm. XRD patterns of the powder samples were taken using a PANalytical’s X’Pert Pro X-ray diffractrometer. During the experiment, Cu K_α_ (λ = 1.54 Å) radiations were used, scan speed was ~0.1 °min^−1^ and step size was ~0.013°. XRD patterns of the heat treated pellets were also taken with similar experimental conditions. Fourier Transform Infrared (FTIR) transmission spectra of the samples in the wavenumber range 4000 cm^−1^–400 cm^−1^ were obtained on Perkin Elmer- Spectrum-RX-I FTIR spectrometer with spectral resolution 0.8 cm^−1^. Powder samples were mixed with KBr for FTIR measurements. Spectrum of the samples was normalized to the spectrum of KBr. Along with FTIR spectroscopy, Raman spectra of the powder samples were also obtained to know the functionality of different molecules within 50–600 cm^−1^ using a Renishaw inVia Raman spectrometer with the 514.5 nm line of an Ar^+^ laser at 20 mW power. Silicon was used as a reference to calibrate the instrument at 520 cm^−1^ within the spectral error of ±1 cm^−1^. Impedance measurements were carried out on SOLARTRON (SI 1260) impedance analyser within a temperature span of 30–500 °C. Powders were pelletized using a 12 kNcm^−1^ pressure applied by the hydraulic press for 3 min (minutes). Pellets were then heat treated at 1000 °C in an electric furnace for 10 hours. These pellets were coated by Pt on both the sides using JEOL Auto Fine Coater (JEC-3000FC) at 20A for 3 min. Measurements were carried out within the frequency and temperature range of 10^2^–10^6^ Hz and room temperature to 400 °C, respectively under air atmosphere. Furnace temperature was increased at a rate of 5 °C min^−1^. At every temperature, a delay of 2 min was imposed for thermal stability of ±1 °C. Scanning electron micrographs of the fractured surfaces of the heat treated pellets were taken on JEOL/EO (version 1.0) SEM after Pt coating on Auto Fine Coater at 20A for 40 sec.

## Additional Information

**How to cite this article**: Danewalia, S. S. *et al*. Agricultural wastes as a resource of raw materials for developing low-dielectric glass-ceramics. *Sci. Rep*. **6**, 24617; doi: 10.1038/srep24617 (2016).

## Figures and Tables

**Table 1:** Composition of the quenched glasses and glass-ceramics from the ICP data.

Sample	SiO_2_	CaO	Na_2_O	MgO	K_2_O	Al_2_O_3_	Fe_2_O_3_	ZnO	CuO
R-100	98.17	0.31	0.32	0.35	0.78	0.17	0.00	0.02	0.00
R-75	93.20	3.91	0.00	1.18	1.44	0.65	0.00	0.01	0.00
R-50	66.51	9.78	0.81	8.66	4.03	18.50	0.55	0.03	0.01
R-25	78.77	7.46	0.75	6.71	3.43	10.30	0.72	0.02	0.01
R-00	78.56	9.91	0.00	6.11	2.74	2.21	0.54	0.03	0.01

**Table 2:** Various structural parameters of the present glasses and glass-ceramics.

Sample	No. of Q_4_ units	No. of Q_3_ units	NBOs	BOs	NBOs (%)
R-100	95.20	3.20	3.20	390.40	0.81
R-75	82.34	11.75	11.75	364.60	3.12
R-50	87.22	8.46	8.46	374.28	2.21
R-25	77.89	14.69	14.69	355.64	3.97
R-00	51.85	32.05	32.05	303.56	9.55
